# Insomnia

**Published:** 1877-01

**Authors:** J. Suydam Knox


### INSOMNIA: ITS CAUSES AND TREATMENT.

BY J. SUYDAM KNOX, M.D.

(Read before the Chicago Medical Society.)

It is undoubtedly true, though we have no direct evidence to that effect, that in the human body functional life means structural death. Muscular force, nerve energy and glandular activity imply molecular disintegration. It is also equally true that, supplementing this waste, there is a constant and corresponding repair. Alternately waste or repair may be in excess; but a mean equilibrium is preserved. This equilibrium is health. Continued excess in either direction is disease.

Now, nutrition being constant, repair will be greatest during periods of the least activity. Taking the amount of the absorption of oxygen and excretion of carbonic acid as a measure of tissue waste, we find that during digestion, muscular exertion, or mental activity, these amounts are greatly increased; during rest both are diminished, and during sleep both are at a minimum.

Sleep, then, is the normal period of greatest repair. As during the waking hours waste exceeds the nutrition, so during sleep repair exceeds disintegration. Sleep, then, is necessary to maintain the equilibrium between integration and disintegration. When development is superadded to nutrition, as in infancy and growing childhood, sleep is prolonged. On the other hand, in old age, when, with decreasing functional activities, progressive decay is overshadowing nutrition, sleep is shortened. The necessity of sleep, therefore, to maintain physical vigor is apparent without argument. And when insomnia, as a resultant, is added to the rapid disintegration and consequent exhaustion of disease, it requires no logic to impress the gravity of this complication upon the physician.

To understand how best to combat insomnia, it is necessary to know the phenomena of healthy sleep. As far as our subjective knowledge goes, during healthy sleep we observe that the brain is in a condition of partial or absolute repose. There is an absence of all voluntary exertion; and though exertion is possible to change a painful position or avoid a cause of irritation, it is made unconsciously, and without awakening; sensibility is blunted, and the special senses inactive; from absence of all voluntary muscular effort, frequency of pulse and blood pressure are diminished, though not more so than during complete repose without sleep. We thus see that sleep is a condition of the nervous system. It is a corroborative fact, that while muscular exhaustion can be overcome by simple rest, nervous exhaustion can only be recuperated by sleep.

Two opposite theories have been advanced to account for the immediate cause of sleep. One attributes it to venous congestion, or increased blood pressure in the brain. This theory is at fault, however, in this respect: that compression of the brain gives rise to stupor or coma, with absolute loss of intellectual power. There is no consciousness, no dreams, no sensibility; in nothing resembling healthy sleep. The other theory originated in the researches of Durham in 1860. Trephining the skulls of dogs, in each case a piece of bone was removed, and a watch glass carefully cemented in its place. In this way the condition of the surface of the brain both awake and asleep could easily be observed. During wakefulness the vessels of the pia mater were moderately distended with blood, and the circulation active. During sleep the circulation was very much diminished, and the brain became pale and retracted. Durham's theory, therefore, was that sleep was caused by anæmia of brain.

Influenced by this theory, Dr. Flemming compressed both carotid arteries in himself and others, and was able, by thus arresting the flow of blood to the brain, to produce immediate and profound sleep. In the wakefulness of infants due to teething or other sources of irritation, the hot bath induces sleep, more by the determination of blood from the head than by the relaxing influences of the immersion. Also the peculiar condition called fainting, to all outward appearances, is directly due to a sudden departure of blood from the head, and in all probability is only a profound sleep thus suddenly induced. The conclusion is the more reasonable when it is observed that the best remedy for syncope is to lower the upper extremity of the body below the horizontal, and thus produce a mechanical return of blood to the brain.

Anæmia of the brain is now generally accepted as the direct cause of sleep, due undoubtedly to vaso motor nerve influence. Positive knowledge can go no further; but observation teaches that long continued physical effort, requiring repose; repose itself; soothing sounds and influences, lulling mental activity; mechanical or sympathetic influences, like position or revulsion, tending to withdraw blood from the encephalon; the removal of light or sound; in fact the withdrawal of all causes that provoke reflex nervous action, are the factors that accomplish this anæmia and lead to sleep. For the cerebrum may be looked upon as an inhibitory influence upon the sympathetic. Withdraw this influence, and the vaso motors successfully exert a tension upon the cerebral capillaries which is constant, though during wakefulness controlled.

Accepting then the theory that sleep is caused by cerebral anæmia, it follows naturally that insomnia arises from cerebral hyperæmia. And since the causes of this hyperæmia are various, as a corollary it may be stated that no one remedy can in all cases act as a hypnotic. Rather is it necessary to differentiate the disturbing element, and remove or neutralize it.

Among the many causes that determine this cerebral hyperæmia, the most prominent are — pain, excitability of afferent nerves or nerve centers, high temperature, and sympathetic derangements of circulation determining to the head. These all admit of various combinations, calling for corresponding variations of treatment.

*Pain.*—Under this head are included all grades of pain, from mere physical discomfort to extreme agony. All pain provokes reflex nerve activity, and thus maintains cerebral hyperæmia. In its milder forms the resulting insomnia is best combatted by the use of the bromides, whose physiological action is to lessen the susceptibility of the afferent nerves and quiet cerebral excitement. In graver forms of pain there is (outside of anæsthetics) no analgesic equal to opium, and it alone can be relied on. Under such circumstances sleep forced by chloral hydrate is restless and unrefreshing; and the doses required of it and potassic bromide are too large to be safely continued.

The salts of morphia are undoubtedly the best form of opium, and the hypodermic syringe the best means for its administration. By this method the first or stimulating influence of the drug is escaped, and the patient passes at once into sleep. Another advantage is gained in avoiding the local benumbing effects of the drug upon the stomach, and the transference of those effects to the seat of pain, by there placing the injection. This latter is well illustrated in the superior influence of suppositories of opium, or rectal injections of some form of it, for pain located in the rectum or genito urinary organs. Various authorities condemn the use of over one-sixth grain of morphia hypodermically; but my observation has been that such an amount is often inefficient. I seldom use for an adult less than from seven to ten minims of Magendie's solution of morphia (16 gr. to ℥j), equal to one-fourth to one-third grain of morphia, and in hundreds of such injections have never seen any bad results.

Owing to the fact that therapeutic doses of opium increase the force of the heart's action and stimulate the nerve centers, it is contra indicated in inflammation or acute congestion of the brain. My own conviction, however, is that opium can often be safely used, if properly guarded, where tradition has forbidden it, provided always that the cause of brain congestion is not permanent. It is often a question whether the extreme reflex nerve irritability and irritative fever growing out of continued severe pain, will not produce more cerebral hyperæmia than a full dose of opium, and whether centric nerve inactivity, with passive congestion, is not preferable to reflex irritability and active congestion. The principal objection is to the stimulating effect of the drug; but with full doses used hypodermically this effect is very transient. Under such circumstances, to neutralize the objectionable influence of the drug, and yet not lose its analgesic power, a hot bath or a hot foot bath should be taken before its administration, or better still, drop doses of tr. aconite root should be given each fifteen minutes until three or five are taken, and the injection then be administered. Another contra-indication to the use of opium is idiosyncrasy. In certain nervous women opium produces extreme nervous prostration, with headache and excessive vomiting. In such cases, where its administration is a necessity, it should be used hypodermically, preceded for twenty minutes by thirty or forty grains of potassic bromide, or it may be given in solution with fifteen or twenty grains chloral hydrate. Either combination diminishes the amount of morphia required, and at the same time lessens the reflex irritability of the nerve centers. As the force of this depressing effect of opium seems to be expended upon the pneumogastric nerves, a minute amount of atropia might be added to each dose, as a stimulant to the vagi, but not enough to antidote the hypnotic effect.

A final contra-indication to the use of opium is the danger of forming the opium habit. In chronic insomnia it should be used with great care. It is a deleterious drug, and has too long been looked upon as the only reliable hypnotic. Physicians are largely responsible that nearly every household, among its domestic remedies, contains a bottle of paregoric for babies and laudanum for adults. The truth is, that as a

*pure* hypnotic, opium should never be used, unless other symptoms or severe pain temporarily demand it.

When pain rises to agony sleep is imperative, both from a humanitarian point of view and as a means of preventing rapid nerve exhaustion. In such cases moderate doses of opium are inefficient, and the only efficient means — anesthesia — cannot be long enough continued. An ingenious combination of the two will often secure long and profound sleep. The method is first to produce complete anesthesia with chloroform or ether, and then to administer hypodermically from $\frac{1}{4}$ to $\frac{1}{2}$ gr. morphia. In this way from eight to fifteen hours of profound sleep may be obtained. Though not sufficiently tested to be proven absolutely innocent, this method has so far been followed by no bad results.

A second cause of insomnia is excitability of afferent nerves or nerve centers. This is the prolific source of almost all wakefulness. Whether we have unrest produced by over mental work or excessive anxiety, whether the emotions or passions enter as factors, or excessive venery, or uterine derangements, or abuse of alcohol, or the specific poisons of disease exist as active causes, the result is the same, viz.: Excessive nervous reflex activity, opposing anæmia of brain, and forbidding sleep. In all such cases treatment of the remote causes is necessary. For the suppression of the immediate cause, the reflex irritability thus produced, two drugs are deservedly held in high esteem, namely, chloral hydrate and potassic bromide.

Chloral hydrate is a pure hypnotic, producing sleep in all points like natural repose. Physiologically it acts directly upon the cerebrum and nerve centers, producing slower pulse, lessened respiration, lowered temperature, and diminished consumption of oxygen and elimination of carbonic acid. In larger doses it produces decided fall of temperature, and progressively paralyzes the nerve centers. Its therapeutic uses are in accord with its physiological effects. In all nervous wakefulness from mental or physical overwork, or of an emotional character, chloral hydrate is indicated. In extreme debility it is forbidden on account of its paralyzing effect upon the nerve centers; and in pain it is useless on account of its slight anæsthetic properties.

On the other hand, potassic bromide is specially useful in nervous wakefulness due to excessive venery; or from sympathy with uterine disorders, or from "unstrung nerves" resulting from abuse of tea, coffee, alcohol or tobacco, or in convalescence from acute disorders. Physiologically it lessens the susceptibility of afferent or sensory nerves, by direct action paralyzing their peripheries. It also quiets cerebral excitement by direct action on the brain centers, and has special control over the genito-urinary nervous system. In large doses it weakens the heart muscle, thus diminishing the force of its beat and the blood pressure, and lowers temperature by retarding tissue change. In doses as large as will be tolerated by the stomach it appears to be harmless, but on account of its weakening effect upon the heart muscle, extreme debility is a contra-indication to its use.

While these two drugs are specially indicated in nervous wakefulness, other symptoms may suggest various combinations. As illustrations — with anæmic and hysterical females the chloral or bromide may be associated with an anti-spasmodic and diffusible stimulant; or in cases due to over mental work or anxiety, accompanied with headache and passive brain congestion, a few drops of the tincture of aconite root, or the fluid extract of gelseminum, or the tincture of veratrum viride may be added to each dose of either medicine. Or mechanical means may be used. Thus in cases where the uterus is the remote cause of the insomnia, a hot vaginal douche, continued for fifteen or twenty minutes, in addition to the bromide, will wonderfully facilitate sleep, provided local conditions do not contra-indicate it.

Still another source of nervous wakefulness is found in extreme physical and nervous exhaustion. Almost every physician has observed the jactitation and delirium after profuse hæmorrhage, or the quiet raving in continued and severe fevers. At first sight wakefulness here would appear paradoxical, for surely with so fluttering a pulse, and so low a blood pressure, anæmia of brain must be excessive. However, the demands from all parts of the system for increased blood supply, or greater innervation, are so imperative that in the brain centers reflex nerve activity is intense, and anæmia as a cause of sleep is completely overwhelmed. While in such cases opium should be heroically administered, still even such use of this drug will be abortive unless accompanied by the free exhibition of diffusible stimulants and nutrition. A good plate of raw oysters will often cause sleep where a proper dose of morphia alone would utterly fail; and brandy can be a godsend to a patient who has been muttering under the influence of opium for hours.

A third cause of insomnia is high temperature. The effect of heat is to stimulate all functional activity. As long as this stimulation is moderate no evil results, for while tissue disintegration is more rapid, integration is correspondingly hastened. If, however, the heat be excessive, destruction surpasses nutrition, and exhaustion begins. In addition, overtaxed construction produces cells, whose only function is to die, and fatty degeneration follows. Thus with exhaustion and deterioration of tissue, the demands upon the nerve centers for a better nutrition become urgent, and an extreme reflex activity is awakened. Still further, the stimulus of great heat creates an intense molecular vibration in the nerves themselves, and reflex activity is converted into an excessive irritability. Wakefulness, delirium, and even mania, are the necessary results. The situation is critical and perplexing. Arterial sedatives are indicated, but the cardiac weakness frowns upon them; depresso-motors seem called for, but they are powerless before the extreme irritability. Three remedies have partially met this condition, namely: quinine, alcohol, and salicylic acid. In large doses they all decidedly lower temperature and retard tissue change. Salicylic acid probably is the best antipyretic known. (? Ed.) Still their success is but partial; and I am fully convinced, after a careful review of cases, that the direct abstraction of heat is the remedy. It is a positive relief to read of the success that has attended the thorough use of the cold bath in Germany, especially as reported by Jürgensen.

The public, and even physicians, have been slow to learn that ice, and abundance of cold drinks, are not injurious in fever, and that fever patients cannot catch cold from free ventilation and scanty covering. They should also be brought to look upon the cold bath as a valuable therapeutic agent, and not a dangerous experiment.

The last cause of insomnia to be considered, namely: determination of blood to the head, while present as a secondary effect in all the causes preceding, is here referred to in a primary sense. Central reflex activity, arising from pain, irritability, or heat, necessitates an increased and active cerebral circulation. Under this head, however, the persistence in the brain of an increased volume of blood, due to chronic derangements of circulation, is referred to. An illustration will explain my meaning, and exemplify the treatment. Practitioners are constantly consulted by patients — most commonly females — whose sleep is fitful and unrefreshing without patent cause. To all outward seeming, their nutrition and innervation is at par. Inquiry, however, develops the fact that they suffer constantly from cold extremities, and periodically from headaches of a congestive character. The underlying cause of their unrest is no doubt in the sympathetic nerve system. The direct cause, however, is an unequal distribution of circulation. In such and kindred cases it would be unwise to direct the medication against the resultant insomnia. Simple logic, unbiased by therapy, would lead to an equalization of blood supply. In many cases this passive brain congestion is in sympathy with some functional derangement. If digestion is at fault (often unrecognized by the patient), it should be corrected. The hours, and quantity and quality of meals, should be regulated. Especially should the supper be exceedingly light, and the dinner eaten near noon. If constipation is habitual, the bowels should be aroused from their torpor, not by purgatives, but by drugs that stimulate their peristaltic action and normal secretions. If the irritation be ovarian or uterine, proper specific treatment should be adopted. Should none of these causes be present, and the maldistribution be apparently due to torpor of the vaso motor nerves, such nerves should be stimulated by mechanical means, such as exercise, the cold douche, frictions, and electrical shampooing. Perseverance in discovering and treating the remote cause will generally be crowned with brilliant success.

The thoughts thus committed to paper are simply suggestions touching upon the subject, and pointing to a wide field for research. The few drugs mentioned are not chosen as specifics, but as the best representatives of a large class of remedies. When they fail there will be time and opportunity enough for a vast selection.

